# Compound A Increases Cell Infiltration in Target Organs of Acute Graft-versus-Host Disease (aGVHD) in a Mouse Model

**DOI:** 10.3390/molecules26144237

**Published:** 2021-07-12

**Authors:** Abdellatif Bouazzaoui, Ahmed A. H. Abdellatif, Faisal A. Al-Allaf, Neda M. Bogari, Mohiuddin M. Taher, Mohammad Athar, Thomas Schubert, Turki M. Habeebullah, Sameer H. Qari

**Affiliations:** 1 Department of Medical Genetics, Faculty of Medicine, Umm Al-Qura University, Makkah 21955, Saudi Arabia; faallaf@uqu.edu.sa (F.A.A.-A.); nmbogari@uqu.edu.sa (N.M.B.); taher23223@yahoo.com (M.M.T.); athar80@gmail.com (M.A.); 2 Science and Technology Unit, Umm Al-Qura University, Makkah 21955, Saudi Arabia; 3 Medical Clinic 3–Hematology/Oncology, University Hospital Regensburg, Franz-Josef-Strauß-Allee 11, 93053 Regensburg, Germany; 4 Department of Pharmaceutics, College of Pharmacy, Qassim University, Qassim 51452, Saudi Arabia; a.abdellatif@qu.edu.sa; 5 Department of Pharmaceutics and Industrial Pharmacy, Faculty of Pharmacy, Al-Azhar University, Assiut 71524, Egypt; 6 Institut für Angewandte Pathologie Speyer, Alter Postweg 1, 67346 Speyer, Germany; schubert@pathospeyer.de; 7 Environment and Health Research Department, The Custodian of the Two Holy Mosques Institute for Hajj and Umrah Research, Umm Al-Qura University, Makkah 21955, Saudi Arabia; tmhabeebullah@uqu.edu.sa; 8 Biology Department, Aljumum University College, Umm Al-Qura University, Makkah 21955, Saudi Arabia; shqari@uqu.edu.sa

**Keywords:** stem cell transplantation, graft-versus-host disease, compound A, bioactive compounds, T cells, cytokines, chemokine, bone marrow transplantation, mouse model

## Abstract

Systemic steroids are used to treat acute graft-versus-host disease (aGVHD) caused by allogenic bone marrow transplantation (allo-BMT); however, their prolonged use results in complications. Hence, new agents for treating aGVHD are required. Recently, a new compound A (CpdA), with anti-inflammatory activity and reduced side effects compared to steroids, has been identified. Here, we aimed to determine whether CpdA can improve the outcome of aGVHD when administered after transplantation in a mouse model (C57BL/6 in B6D2F1). After conditioning with 9Gy total body irradiation, mice were infused with bone marrow (BM) cells and splenocytes from either syngeneic (B6D2F1) or allogeneic (C57BL/6) donors. The animals were subsequently treated (3 days/week) with 7.5 mg/kg CpdA from day +15 to day +28; the controls received 0.9% NaCl. Thereafter, the incidence and severity of aGVHD in aGVHD target organs were analyzed. Survival and clinical scores did not differ significantly; however, CpdA-treated animals showed high cell infiltration in the target organs. In bulk mixed lymphocyte reactions, CpdA treatment reduced the cell proliferation and expression of inflammatory cytokines and chemokines compared to controls, whereas levels of TNF, IL-23, chemokines, and chemokine receptors increased. CpdA significantly reduced proliferation in vitro but increased T cell infiltration in target organs.

## 1. Introduction

Allogeneic bone marrow transplantation (allo-BMT) is a curative therapy for diverse hematological and malignant diseases. However, its utility is limited due to the development of severe treatment-related complications, especially acute graft-versus-host-disease (aGVHD), which is the major cause of morbidity and mortality after allogeneic stem cell transplantation (allo-SCT) [1,2,3]. Hence, the ability to control transplantation-related complications, including aGVHD, remains an essential goal in improving allo-BMT outcomes. Owing to their potent immunosuppressive and anti-inflammatory properties [4], glucocorticoids (GCs) are the most effective for treating various inflammatory/autoimmune disorders. They have been used to treat different inflammatory diseases such as asthma, inflammatory bowel disease, rheumatoid arthritis, and other diseases [5,6,7,8,9,10,11]. Furthermore, GCs are used as immunosuppressive drugs in organ transplantation and hematological malignancies [12,13]. For more than 30 years, systemic GC administration has been the standard treatment for aGVHD [14,15,16,17,18,19,20]; however, the use of GCs did not decrease the complications associated with aGVHD prophylaxis and the frequency of severe aGVHD occurrence [21,22,23,24]. Furthermore, prolonged application of steroids results in severe metabolic side effects, including diabetes, fat dysregulation, skin disorders, muscle atrophy, and alteration of behavior [13,25].

The effects of steroids are mediated via the glucocorticoid receptor (GC) [26], localized in the cytoplasm in its inactivated form. GCs penetrate the cell, bind to the GR, and cause conformational changes; thereafter, the GC-GR complex migrates to the nucleus and activates different genes [27,28]. The side effects are believed to mainly occur due to the transactivation of GR [25,29]. Consequently, GR ligands that unlink transrepression from transactivation prohibit dimerization of GRs [30,31] and may preserve therapeutic activity with fewer side effects. Recently, researchers characterized compound A (CpdA) [2-((4-acetoxyphenyl)-2-chloro-n-methyl) ethylammonium chloride], a synthetic analog of a natural compound discovered in *Salsola tuberculatiformis Botschantzev*, responsible for the prolonged gestation in Karakul sheep in Namibia and the Republic of South Africa [32]. Previous studies have demonstrated the strong anti-inflammatory activity and minimal adverse effects of CpdA [30,33]. In addition, a recent study has demonstrated that the exposure of immune cells to CpdA inhibits the expression of pro-inflammatory genes [34,35]. Furthermore, experimental models have revealed that CpdA reduces inflammatory disorders [5], autoimmune neuritis and encephalomyelitis [6,7,36], arthritis [8,11,30], asthma [10], and inflammatory bowel disease [9].

aGVHD is characterized by the induction of inflammatory cytokines, chemokines, and chemokine receptors and the subsequent activation, proliferation, and migration of T cells to the target organs. The migration of T cells, in turn, affects the expression of inflammatory mediators involved in target organ infiltration and injury [37,38,39,40,41,42,43,44]. Hence, this study aimed to assess the effects of CpdA in a preclinical aGVHD mouse model to introduce a new agent for the treatment of aGVHD.

## 2. Results

### 2.1. Influence of CpdA on Proliferation and Inflammation In Vitro

**Figure 1:**
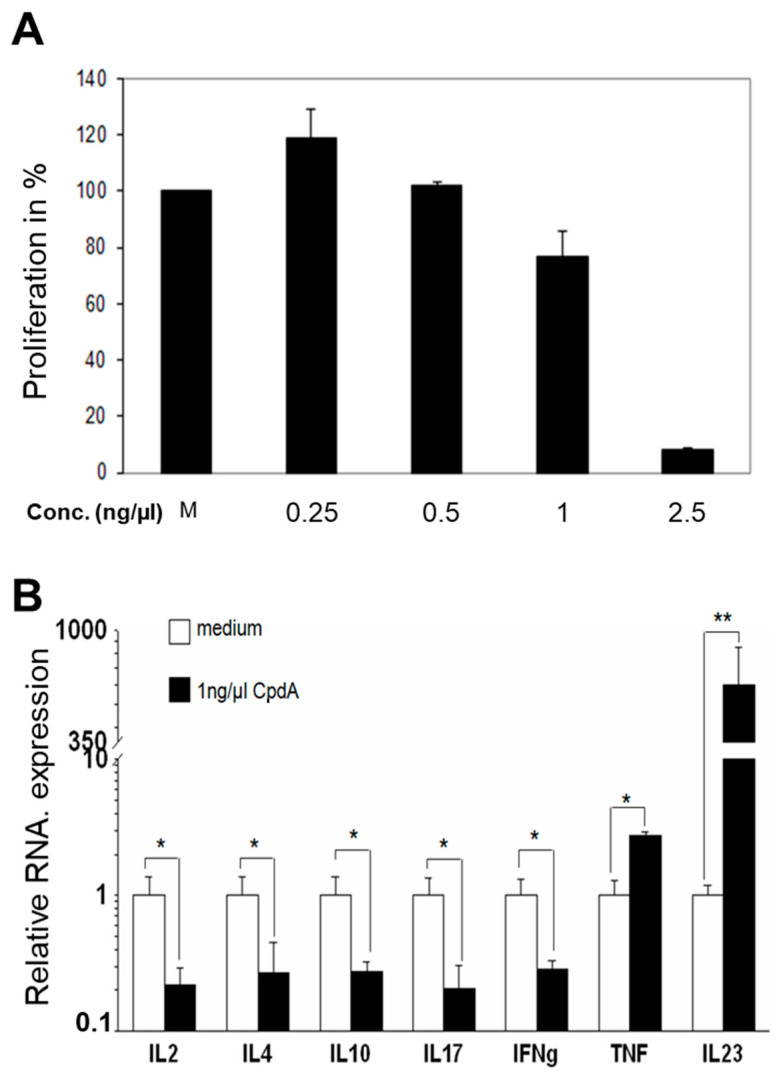
Mixed lymphocyte reaction (MLR) proliferation assay and cytokine expression. The MLR was performed as described in the Section 4. (**A**) T cell proliferation in response to alloantigen stimulation with different doses of CpdA. (**B**) mRNA expression levels in cells measured using real-time PCR after the MLR. CpdA concentration indicates the amount added during bulk MLR. The combined results from the two experiments are shown, and the data are presented as mean ± SEM; * *p* < 0.05, ** *p* < 0.01.

Based on the results of previous studies demonstrating strong anti-inflammatory responses and reductions in pro-inflammatory gene expression after treatment with CpdA [30,33], we analyzed the effect of CpdA on T cell proliferation using the mixed lymphocyte reaction (MLR) assay. The analysis revealed lesser proliferation of CpdA-treated cells than the control cells. Compared to the control, the proliferation reached 76%, presenting a reduction of 24% when the cells were treated with 1 ng/µL CpdA, and the proliferation was almost completely suppressed (only 8% proliferating cells) when cells were treated with 2.5 ng/µL CpdA (Figure 1A). The relative expression levels of IL-2, IL-4, IL-10, IL-17, and IFNγ in the treated samples were significantly lower than in control samples; however, TNF and IL-23 expression was significantly higher in CpdA-treated samples than in control samples (Figure 1B).

### 2.2. Effect of CpdA on Allo-BMT In Vivo

**Figure 2:**
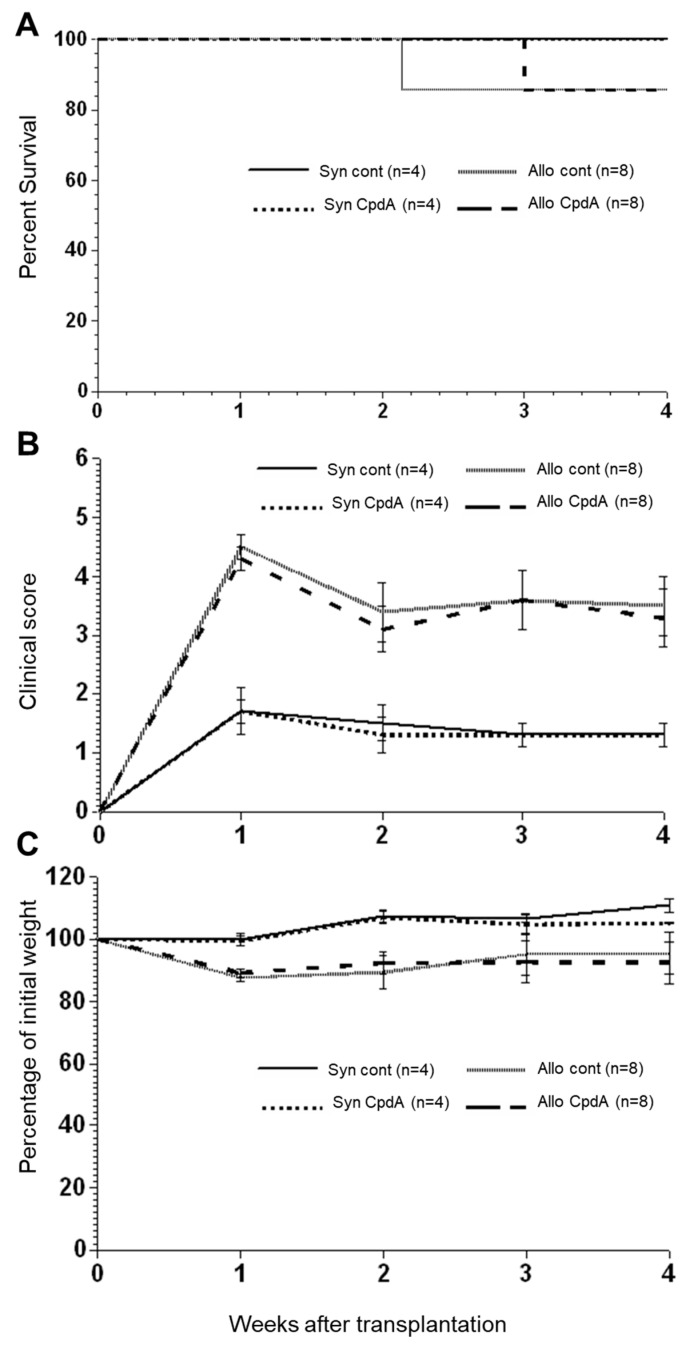
Treatment of model mice with CpdA after allo-BMT. Lethally irradiated B6D2F1 mice were transplanted as described in the Section 4 and treated intraperitoneally thrice a week with 150 µg of CpdA from day +15 to day +28. The control animals received 0.9% NaCl. The diagram shows survival (**A**), clinical scores (**B**), and weight loss (**C**) for syngeneic + NaCl, syngeneic + CpdA-treated, allogeneic + NaCl, and allogeneic + CpdA-treated recipients. Combined results from two experiments are shown, and the data are presented as mean ± SEM.

We analyzed the effect of CpdA in an experimental aGVHD murine model. After transplantation and treatment, the syngeneic recipients showed only minor changes due to radiation toxicity at 1 week after BMT (Figure 2) but continuously recovered and gained weight from week 2 to week 4 after transplantation, and all animals survived until day 28 (Figure 2). The allogeneic recipients had high clinical scores within the first week after transplantation. After that, both the control and CpdA-treated animals partially recovered by week 2, followed by a slight increase in disease severity during the last 2 weeks after transplantation. Interestingly, a comparison of clinical GVHD scores between CpdA-treated and control animals indicated that CpdA treatment did not affect clinical GVHD scores, including the weight of allogeneic mice.

### 2.3. Cytokine Expression and Pathology Score

**Figure 3:**
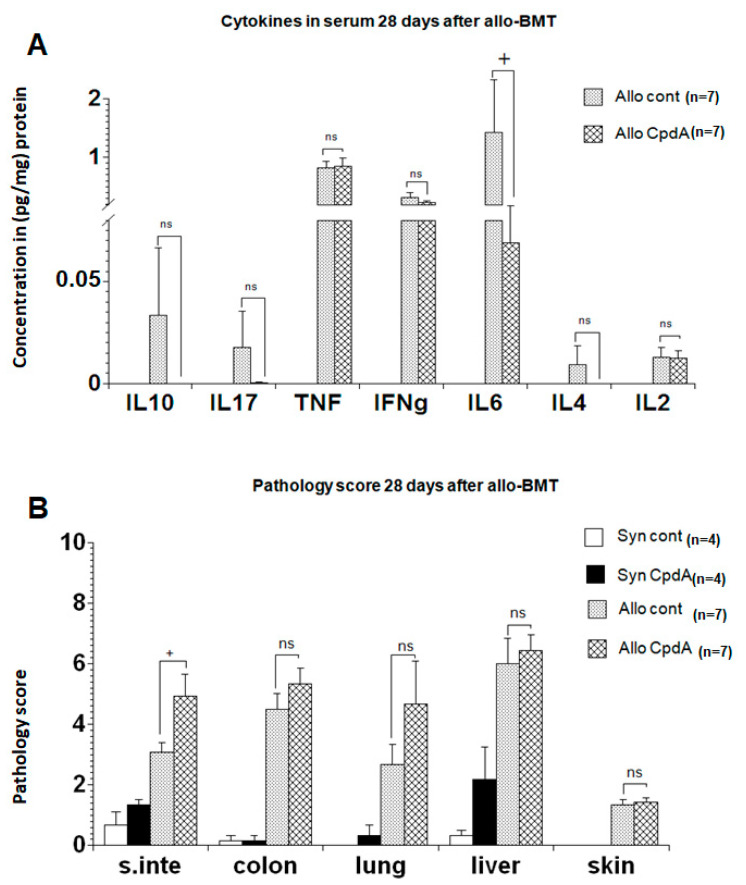
Cytokine expression and pathology score. Lethally irradiated B6D2F1 mice were transplanted as described in the Section 4 and treated intraperitoneally thrice a week with 150 µg of CpdA from day +15 to day +28. The control animals received 0.9% NaCl. (**A**) At day +28 post-transplantation, cytokine levels in serum were determined using CBA. (**B**) Histopathology scores for the small intestine (s.inte), colon, lung, liver, and skin at day +28 after BMT. The combined results from the two experiments are shown, and the data are presented as mean ± SEM; non-significant (ns), tendence to significance (+).

To replicate the initial experiment results regarding survival, clinical scores, and weight changes, the mice were transplanted as described, treated with CpdA or NaCl at day 28 post-transplantation, and then sacrificed. Thereafter, the levels of TNF, IFNγ, IL-2, IL-4, IL-6, IL-10, and IL-17 were determined using a cytokine bead assay (CBA), followed by histopathology of the gastrointestinal tract, liver, lung, and skin. Slight reductions were observed in IFNγ, IL-6, and IL-4 levels in CpdA-treated animals; however, the changes in cytokine levels in the serum were not significant (Figure 3A). In contrast, the pathology scores of the organs in CpdA-treated animals were slightly higher than those in the controls (Figure 3B).

### 2.4. Analysis of Cell Suspensions of Target Organs

**Figure 4:**
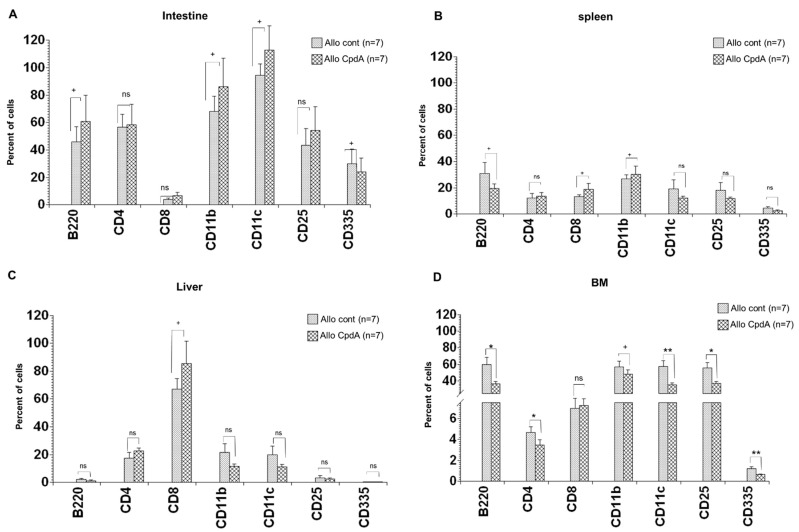
Quantification of cells in target organs. Lethally irradiated B6D2F1 mice received 2 × 10^6^ BM cells supplemented with 4 × 10^6^ splenocytes from either syngeneic (B6D2F1) or allogeneic (C57BL/6) donors and 7.5 mg/kg CpdA intraperitoneally thrice a week from day +15 to day +28. At day +28, the percentages of B220+ cells and CD25+, CD335+, CD11b+, CD11c+, CD4+, and CD8+ T cells in the intestine (**A**), spleen (**B**), liver (**C**), and BM (**D**) were analyzed. The combined results from the two experiments are shown, and the data are presented as mean ± SEM; * *p* < 0.05, ** *p* < 0.01, non-significant (ns), tendence to significance (+).

The spleen, liver, intestine, and BM were harvested at 28 days post-transplantation, and single-cell suspensions were obtained and analyzed as described in the Materials and Methods section. Slightly higher infiltration of all analyzed cell types, including B cells (B220), CD4+ and CD8+ T cells, APCs (CD11b and CD11c), and CD25+ T cells, was observed in the intestine of CpdA-treated animals than in the control mice (Figure 4). In contrast, the number of NK cells (CD335+) was reduced. There was an increase in CD4+ and CD8+ T cell populations in the liver of CpdA-treated animals, whereas there was a decrease in the remaining cell subpopulations. Furthermore, the cell composition in the spleen of CpdA-treated animals showed the same trend as in the liver, except for CD11b+ cells, which showed a slight increase. Interestingly, opposite results were observed in the BM of CpdA-treated animals. Except for CD8+ T cells, the percentage of all other cells was reduced, and the reduction was significant for B cells, CD4+ T cells, CD11c+ APCs, CD25+ T cells, and NK cells.

### 2.5. Expression of Chemokines and Chemokine Receptors after MLR

**Figure 5:**
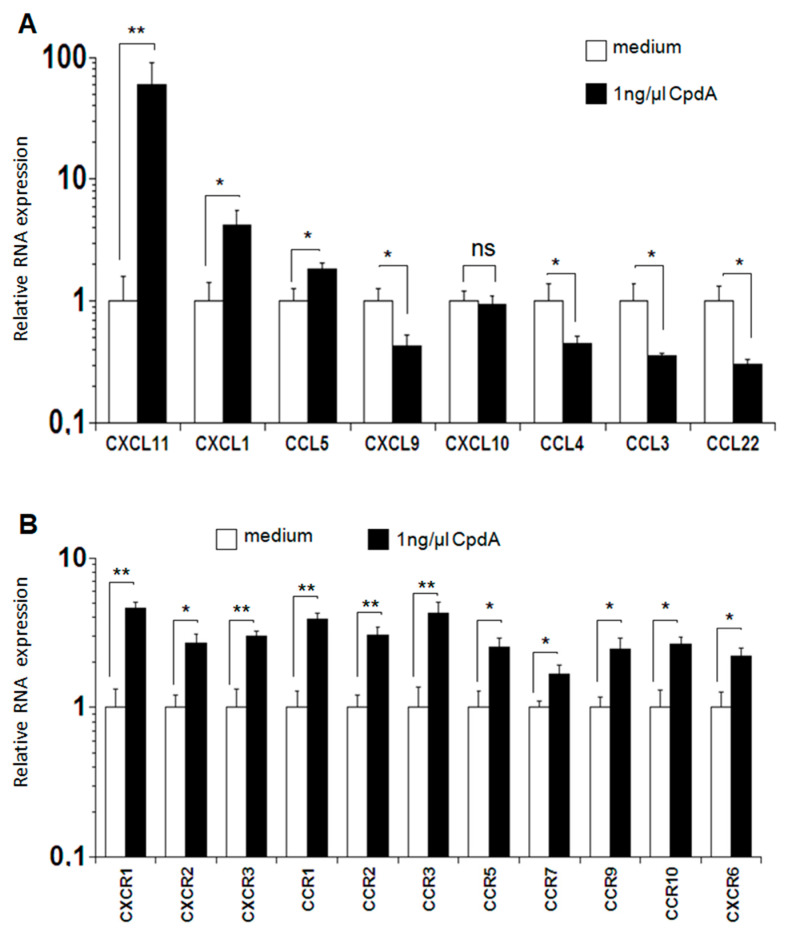
Expression of chemokines and chemokine receptors in cells after MLR. MLR was performed as described in the Section 4. (**A**) mRNA expression levels of chemokines and (**B**) chemokine receptors were determined in cells using real-time PCR at the end of MLR. The combined results from the two experiments are shown, and data are presented as mean ± SEM; * *p <* 0.05, ** *p <* 0.01, non-significant (ns).

The expression of chemokines and chemokine receptors in T cells treated with 1 ng/µL CpdA was analyzed to explain the accumulation of cells in the target organs in vivo. This concentration was selected based on the results of the proliferation assay. After performing MLR in vitro, RNA was isolated, and the expression levels of chemokines and chemokine receptors were analyzed using real-time PCR. The expression levels of the chemokines CXCL11, CXCL1, and CCL5 were higher in CpdA-treated cells than in the controls, with the difference highly significant for CXCL11 and significant for CXCL1 and CCL5. The levels of the spinet chemokine CXCL10 in CpdA-treated samples and controls were similar; however, the expressions of CXCL9, CCL3, CCL4, and CCL22 were significantly lower in CpdA-treated cells than in control cells (Figure 5A). Interestingly, the expression levels of the chemokine receptors CXCR1-3, CXCR6, CCR1-3, CCR5, CCR7, and CCR9-10 were significantly or highly significantly increased in cells treated with CpdA compared to the corresponding levels in control cells (Figure 5B).

## 3. Discussion

Allogeneic bone marrow transplantation (allo-BMT) is a curative therapy for diverse hematological and malignant diseases. However, its utility is curtailed by the development of severe treatment-related complications, especially acute graft-versus-host-disease (aGVHD). In the early work of Wüst et al., the authors found that treating mice with different doses of CpdA led to completely different results depending on CpdA concentration [6]. In an experimental autoimmune encephalomyelitis (EAE), a multiple sclerosis (MS) model, the authors found that the administration of 15 mg/kg CpdA aggravated the disease rather than having a beneficial effect, and was lethal for mice. However, the application of 5 mg/kg and 1.5 mg/kg CpdA significantly ameliorated the disease [6]. Using the same model, van Loo et al. found that treating mice with 150 µg CpdA in PBS, both early and at the disease peak, markedly suppressed the clinical symptoms of EAE induced by myelin oligodendrocyte glycoprotein peptide immunization [7]. Furthermore, CpdA inhibits pro-inflammatory mediators in T cells and reduces cytokine expression, including INFγ and IL-17, inducing apoptosis in various cell types [6,7]. The effect of CpdA requires the expression of GR in T cells via IL-17 repression and the downregulation of LFA-1 and CD44 in peripheral Th cells [6]. Based on the early study from van Loo et al., and to explore whether the new CpdA can improve the outcome of aGVHD, we assessed the effects of CpdA as a treatment alternative for aGVHD in a murine model. In the beginning, we started the analysis using Bulk MLR in vitro. The results indicate a significant reduction in the proliferation capacity of CpdA-treated cells (8% compared to that of control using 2.5 ng/µL CpdA) (Figure 1A) and in the relatively low expression levels of IL-2, IL-4, IL-10, IL-17, and IFNγ, all of which are produced mainly by T cells.

For the in vivo assay using an allo-BMT mouse model, and even though we use the same dose as in previous work [7], CpdA did not show any clinical activity in terms of clinical GVHD scores. In addition, the expression levels of the cytokines IFNγ, IL-6, and IL-4 in CpdA-treated animals were only slightly lower than in control animals. This discrepancy between the effect in vitro and in vivo could be explained by the dose we used to treat animals. It is possible that the application of 150 µg, which is equivalent to 7.5 mg/kg, is excessive for this model. This is supported by the findings by Wüst et al. showing that the application of a high dose (15 mg/kg) of CpdA aggravated the disease rather than having a beneficial effect, and it was lethal for mice. Interestingly, in our experiment, we found that the organs of CpdA-treated animals presented with slightly higher pathology scores than the organs of the control animals, which is in line with the results from Wüst et al.

To explain the increased organ injury in CpdA-treated animals, we referred to previous publications showing the impact of the activation, proliferation, and migration of T cells in the organs as a vital factor in aGVHD development [39,42,44,45]. The increased migration of these cells in target organs correlates with the high production of chemokines and cytokines, ultimately resulting in serious organ injury [39]. In our study, we also observed a higher cell migration in aGVHD target organs, especially CD8+ T cells, which can explain the slightly higher pathology scores in CpdA-treated animals than in control animals.

Moreover, we observed a significant increase in TNF and IL-23 levels in CpdA-treated samples compared to controls in the MLR assay. IL-23, produced mainly by activated APCs including dendritic cells (DCs) [46], consists of two subunits, a small p19 subunit unique to this cytokine and a p40 subunit shared with IL-12. A previous study demonstrated that IL-23 production by DCs is negatively regulated by protein phosphatase 2A (PP2A) [46]. IL-23 expression increased after the downregulation of PP2A, induced by siRNA or okadaic acid treatment. In addition to PP2A, okadaic acid strongly inhibited protein phosphatase 1 via the dephosphorylation of serine and threonine residues [46]. Another study investigated the role of IL-12 p40 in TNF expression and revealed that the p40 homodimer and p40 monomer induced the production of *TNF* mRNA in microglial cells and macrophages [47], leading to the hypothesis that CpdA potentially increases IL-23 by inhibiting PP2A expression via the NF-κB pathway, which may explain the higher TNF expression.

The in vitro analyses of chemokine and chemokine receptor expression indicates significant reductions in the levels of CXCL9, CCL3, CCL4, and CCL22 in cells treated with CpdA compared to those in control cells, which is in agreement with the results of previous studies, demonstrating that exposure of immune cells to CpdA inhibits pro-inflammatory gene expression [34,35].

However, the expression levels of CXCL11, CXCL1, and CCL5 increased significantly in CpdA-treated cells compared to corresponding levels in control cells (Figure 5A). Furthermore, the expression levels of CXCR1-3, CXCR6, CCR1-3, CCR5, CCR7, and CCR9-10 are significantly or highly significantly increased in cells treated with CpdA compared to the levels in control cells (Figure 5B), which may explain the increased recruitment of CD8+ T cells. The treatment with CpdA stimulates the expression of chemokine receptors, which increase the migration of cells in the target organs, resulting in high levels of organ injury. An additional mechanism that may be involved in the observed effect of CpdA is based on IL-23 expression. Previously, Khader et al. have demonstrated that IL-23 positively affects CD4+ T cell migration in the lungs of wild-type mice compared to IL-23 deficient mice [48]. They further indicated that targeting IL-23 triggered the expression of genes encoding CXCR3 and CXCL11. The chemokine receptor CXCR3 is rapidly induced in naïve T cells, and its expression remains high in both CD4+ and CD8+ T cells after activation [49].

## 4. Materials and Methods

### 4.1. Transplantation Procedure

Transplantation was performed as described previously [50]. Briefly, female C57BL/6 (H-2^b^) and B6D2F1 (H-2^bxd^) mice were purchased from Charles River Laboratories (Sulzbach, Germany) and acclimatized to the animal facility for at least 1 week before starting the experiments. The mice were housed in micro-isolator cages with autoclaved bedding and received sterilized chow and water.

The mice were 11–14 weeks old at the time of BMT. The transplantation was conducted as previously described [50]. In summary, on the day of transplantation (day 0), the B6D2F1 recipient mice received a total body irradiation (TBI) dose of 9 Gy delivered in one fraction using a linear accelerator (150 cGy/min). Thereafter, the animals were infused with 2 × 10^6^ BM cells supplemented with 4 × 10^6^ splenocyte cells (SCs) from either syngeneic (B6D2F1 = syn) or allogeneic (C57BL/6 = allo) donors. Based on the results of previous studies from van loo et al. [7], the animals received 150 µg of CpdA (Enzo Life Sciences, Lörrach, Germany) or 0.9% NaCl (control) intraperitoneally thrice a week from day +15 to +28 after transplantation. The survival of the animals was monitored daily, and the clinical GVHD scores were assessed weekly, as previously described [50], and based on the assessment of five clinical parameters: weight loss, activity, posture (hunching), skin integrity, and fur texture, as described previously [51]. On day +28 post-transplantation, the animals were sacrificed, the histopathology of all organs was analyzed, and the cytokine concentrations in serum were determined. Furthermore, the percentage of B220+, CD25+, CD335+, CD11b+, CD11c+, CD4+, and CD8+ T cells in the spleen, liver, intestine, and BM were analyzed.

### 4.2. Semiquantitative Histopathology of the Liver, Lung, Gastrointestinal Tract, and Skin

Following the sacrifice of the mice, the histopathology of the organs was analyzed. Hematoxylin and eosin-stained organ sections from individual mice were coded without reference to mouse type and prior treatment. A detailed analysis of the changes in the gastrointestinal tract, liver, lung, and skin histology was performed in a blinded fashion as described previously [39,50,52,53,54]. In summary, a semiquantitative scoring system has been used to analyze the following changes associated with aGVHD development: small intestine and colon—luminal sloughing of cellular debris, villous blunting, loss of enterocyte brush border, surface colonocytes, colonocyte vacuolization, surface colonocyte attenuation, crypt regeneration, outright crypt destruction, crypt cell apoptosis, and lamina propria lymphocytic infiltrate [54]; liver and lunge—lobular changes, expansion of portal triads, hepatocellular changes, architectural distortion, cholestasis, inflammation in triads, lymphocytic infiltrate, and injury to interlobular bile ducts [55]. The scoring system denoted 4.0 as diffuse and severe, 3.0 as diffuse and moderate, 2.0 as diffuse and mild, 1.0 as focal and mild, 0.5 as focal and rare, and 0 as normal. Finally, scores were added to build a total score for each animal.

### 4.3. Serum Cytokine Analysis after BMT

The animals were exsanguinated at +28 days after BMT. Blood samples were collected in 1.5 mL Eppendorf tubes (Hamburg, Germany) and centrifuged at 10,000 rpm for 5 min. Serum levels of TNF, IFNγ, IL-2, IL-4, IL-6, IL-10, and IL-17 were determined using CBA kits from BD Biosciences (Heidelberg, Germany) according to the manufacturer’s protocol. CBA beads were analyzed using BD FACSCalibur (BD Biosciences, Heidelberg, Germany), and the results were analyzed using the FCAP Array v3.0 and normalized to protein concentration.

### 4.4. Single-Cell Suspensions and Flow Cytometry Analysis

At day +28 post-transplantation, the spleen, liver, intestine, and BM were harvested, and single-cell suspensions were obtained using the gentleMACS^TM^ dissociator and kits from Miltenyi Biotec (Bergisch Gladbach, Germany) according to the manufacturer’s protocol. The cells were then analyzed for H-2k^b^, H-2k^d^ and CD4+ (T cells), CD8+ (T cells), CD3+ (T cells), CD25+ (T cells), B220+ (B cells), CD11b+ (macrophages), CD11c+ (dendritic cells), and CD335+ (NK cells) expression using the following antibodies: fluorescein isothiocyanate (FITC)-conjugated mouse anti-mouse-H-2k^b^, phycoerythrin (PE)-conjugated mouse anti-mouse-H-2k^d^, V500-conjugated rat anti-mouse-CD4, antigen presenting cell (APC)-conjugated rat anti-mouse-CD8, Pacific Bl-conjugated Syrian hamster anti-mouse-CD3, APC-Cy7-conjugated rat anti-mouse-CD25, PE-Texas Red-conjugated rat anti-mouse-B220, Alex Fluor^®^ 700-conjugated rat anti-mouse-CD11b, PE-Cy7-conjugated hamster anti-mouse-CD11c, and PerCP-Cy5.5-conjugated rat anti-mouse-CD335. After blocking with 4% FCS in PBS, we used H-2k^b^-FITC to define the donor phenotype and the gating region from which we define the percentage of the different markers; the control samples were labeled with isotype antibodies (see Supplementary Materials). All monoclonal antibodies were purchased from BD Pharmingen (Heidelberg, Germany), and flow cytometric analysis of 5 × 10^5^ cells was performed using the LSR2 instrument from BD Biosciences. The results were analyzed using FlowJo version 9.3.3 from Tree Star (Ashland, OR, USA).

### 4.5. T Cell Proliferation and In Vitro Mixed Lymphocyte Reaction (MLR)

All cell culture reagents were purchased from Invitrogen (Carlsbad, CA, USA) or Sigma-Aldrich (Taufkirchen, Germany). The cell cultures were maintained at 37 °C in a humidified incubator in the presence of 5% CO_2_. CD90+ T cells were isolated via magnetic bead separation using the QuadroMACS system (Miltenyi Biotec, Bergisch Gladbach, Germany), according to the manufacturer’s protocol. T cell proliferation in response to alloantigen was determined by co-culturing 2 × 10^5^ CD90+ T cells from C57BL/6 mice in 96-well flat-bottom plates with 4 × 10^5^ irradiated (30 Gy) B6D2F1 splenocytes in the presence of different concentrations of CpdA (0.25–2.5 ng/µL). Proliferative responses were assessed via MLR, using a Topcount microplate scintillation counter (Packard Canberra, Dreieich, Germany) in terms of [^3^H] thymidine incorporation (1.9 × 10^5^ Bq/mL) for the last 16 h of a 48h incubation. CD90+ T cells were stimulated with 2.5 µg/mL concanavalin A (Sigma, St. Louis, MO, USA) as a positive control for assessing the viability and proliferative capacity of T cells.

### 4.6. Determination of mRNA Expression Using Real-Time PCR

As the proliferation assay demonstrated that 1 ng/µL CpdA reduced the proliferation to 76%, this concentration was selected for mRNA expression analysis. After treatment, total mRNA was extracted using the RNA Miniprep Kit (Sigma-Aldrich, Taufkirchen, Germany), according to the manufacturer’s protocol. First strand cDNA was synthesized using 1 µg of total RNA (DNase-treated) and the Maxima first strand cDNA synthesis kit (Thermo Fisher Scientific, Waltham, MA, USA) and stored at −20 °C until use. All real-time PCRs were performed according to a standard protocol as described previously [39,56]. Quantitative real-time PCR was performed using the ABI PRISM^®^ 7900HT sequence detection system from Applied Biosystems (Foster City, CA, USA). The fluorescence threshold value was calculated using ABI PRISM^®^ 7900HT sequence detection system software (version 2.2); the housekeeping gene β-actin was used for normalization. The primers used for real-time PCR are listed in Table 1.

### 4.7. Statistical Methods

Pathology scores, expression levels of cytokines, relative RNA expression, and the percentage of cells in the organs were compared between CpdA-treated animals and controls at individual time points using a nonparametric unpaired Mann–Whitney U test. Statistical significance and high significance for all analyses were set at *p* < 0.05 and *p* < 0.01, respectively. Data were presented as the mean ± standard error of the mean (SEM). Kaplan–Meier and log-rank tests were used to analyze survival data. The statistical program SPSS version 22 (IBM, Chicago, IL, USA) was used for analysis.

## 5. Conclusions

Our results demonstrate the importance of cell migration in aGVHD. Despite the antiproliferative capacity of CpdA, aGVHD symptoms were not ameliorated due to an increase in T cell homing in the affected organs, indicating the impact of cell recruitment on the outcome of aGVHD. For significant improvements in aGVHD, it is necessary to combine CpdA with agents targeting IL-23, such as tremfya or Skyrizi, in future studies.

## Figures and Tables

**Table 1:** Primers used for real-time PCR.

β-Act-sense	GCT GTC CCT GTA TGC CTC TG
β-Act-antisense	GTG GTG GTG AAG CTG TAG CC
CCL3(MIP-1α)-sense	TGC CTG CTG CTT CTC CTA CA
CCL3(MIP-1α)-antisense	TGG ACC CAG GTC TCT TTG GA
CCL4(MIP-1β)-sense	CCA GGG TTC TCA GCA CCA A
CCL4(MIP-1β)-antisense	GCT CAC TGG GGT TAG CAC AGA
CCL5 (RANTES) sense	CTCACCATATGGCTCGGACA
CCL5 (RANTES) antisense	CTTCTCTGGGTTGGCACACA
CXCL1(KC) sense	GCCTATCGCCAATGAGCTG
CXCL1(KC) antisense	CTGAACCAAGGGAGCTTCAGG
CXCL9(MIG)-sense	TGG GCA TCA TCT TCC TGG AG
CXCL9(MIG)-antisense	CCG GAT CTA GGC AGG TTT GA
CXCL10(IP-10)-sense	CCT CAT CCT GCT GGG TCT G
CXCL10(IP-10)-antisense	CTC AAC ACG TGG GCA GGA
CXCL11(I-TAC)-sense	CGG GAT GAA AGC CGT CAA
CXCL11(I-TAC)-antisense	TAT GAG GCG AGC TTG CTT GG
CCL22 (MDC) sense	TGGCTCTCGTCCTTCTTGCT
CCL22 (MDC) antisense	AGGCTTGCGGCAGGATTT
IFN γ-sens	CAG GCC ATC AGC AAC AAC AT
IFN γ-antisens	CGC TTC CTG AGG CTG GAT T
TNF-sens	TAC GTG CTC CTC ACC CAC AC
TNF-antisens	AGT TGG TCC CCC TTC TCC AG
CCR1 sense	TTCCTCCTCTGGACCCCCTA
CCR1 antisense	TTGAAACAGCTGCCGAAGGT
CCR2 sense	CCACACCCTGTTTCGCTGTA
CCR2 antisense	TGCATGGCCTGGTCTAAGTG
CCR3 sense	TTTGGACCCCGTACAACCTG
CCR3 antisense	TTTCCGGAACCTCTCACCAA
CCR5 sense	CAGGGCTGTGAGGCTCATCT
CCR5 antisense	GGCAGCAGTGTGTCATTCCA
CCR7 sense	CCAATAGCAGCTGCGAAACC
CCR7 antisense	GCAGCCCAAGTCCTTGAAGA
CCR9 sense	GGTCACCTTGGGGTTTTTCC
CCR9 antisense	TAAGCGTCAACAGCCTGCAC
CCR10 sense	CCAGCAAGCGCAAGGATCTA
CCR10 antisense	AGCAGGAAGAAAGGCGGAGT
CXCR1 sense	TGGGGGTGATCTTTGCTGTT
CXCR1 antisense	TTTGGCCAACGAAGGCATAG
CXCR2 sense	CATCTTCGCTGTCGTCCTTGT
CXCR2 antisense	GCTGTGGAGGAAGCCAAGAA
CXCR3 sense	TAGTGGTGGTGGCAGCCTTT
CXCR3 antisense	AGGCATAGAGCAGCGGATTG
CXCR6 sense	CTTTCGGGCTTGCCTTAACC
CXCR6 antisense	CATTGTGGGAGGCAGAACAA
IL23a sense	GCCCCGTATCCAGTGTGAAG
IL23a antisense	CTGGGCATCTGTTGGGTCTC
IL17a sense	TCCACGTCACCCTGGACTCT
IL17a antisense	CCCACCAGCATCTTCTCGAC
IL2 sense	GGACCTCTGCGGCATGTT
IL2 antisense	TCTCCTCAGAAAGTCCACCACA
IL4 sense	GATGTGCCAAACGTCCTCAC
IL4 antisense	AAGCCCGAAAGAGTCTCTGC
IL10 sense	GGGTTGCCAAGCCTTATCG
IL10 antisense	TGCTCCACTGCCTTGCTCT

## Data Availability

All data generated and/or analyzed during this research study are available from the corresponding author on reasonable request.

## Supplementary Materials

Supplementary Materials are available online.

## Sample Availability

Samples of the compounds are commercially available.
