# Supplementary material for: Compound A Increases Cell Infiltration in Target Organs of Acute Graft-versus-Host Disease (aGVHD) in a Mouse Model
Source: Molecules. 2021 Jul 12;26(14):4237. doi: 10.3390/molecules26144237 (PMC8303851; doi:10.3390/molecules26144237)

Article

Compound A Increases Cell Infiltration in Target Organs of Acute Graft-Versus-Host Disease (aGVHD) in a Mouse Model

Abdellatif Bouazzaoui [1,2,3,*], Ahmed A. H. Abdellatif [4,5], Faisal A. Al-Allaf [1], Neda M. Bogari [1], Mohiuddin M. Taher [1,2], Mohammad Athar [1,2], Thomas Schubert [6], Turki M. Habeebullah [7] and Sameer H. Qari [8]

[1] Department of Medical Genetics, Faculty of Medicine, Umm Al-Qura University, 21955 Makkah,
Saudi Arabia; faallaf@uqu.edu.sa (F.A.A.-A.); nmbogari@uqu.edu.sa (N.M.B.); taher23223@yahoo.com (M.M.T.); athar80@gmail.com (M.A.)

[2] Science and Technology Unit, Umm Al-Qura University, 21955 Makkah, Saudi Arabia

[3] Medical Clinic 3–Hematology/Oncology, University Hospital Regensburg, Franz-Josef-Strauß-Allee 11, 93053 Regensburg; Germany

[4] Department of Pharmaceutics, College of Pharmacy, Qassim University, 51452 Qassim, Saudi Arabia;
a.abdellatif@qu.edu.sa

[5] Department of Pharmaceutics and Industrial Pharmacy, Faculty of Pharmacy, Al-Azhar University, 71524 Assiut, Egypt

[6] Institut für angewandte Pathologie Speyer, Alter Postweg 1, 67346 Speyer, Germany; schubert@pathospeyer.de

[7] Environment and Health Research Department, The Custodian of the Two Holy Mosques Institute for Hajj and Umrah Research, Umm Al-Qura University, 21955 Makkah, Saudi Arabia;
tmhabeebullah@uqu.edu.sa

[8] Biology Department, Aljumum University College, Umm Al-Qura University, 21955 Makkah, Saudi Arabia; shqari@uqu.edu.sa

* Correspondence: ab1971@hotmail.de or alazzauoi@uqu.edu.sa; Tel.: +966571297636

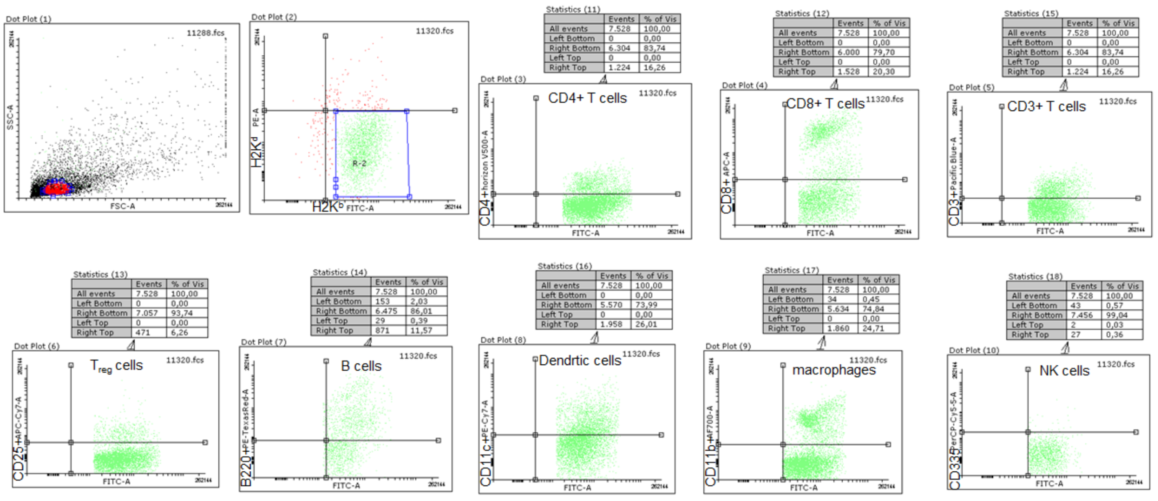

Supplement: Supplementary file 1 [file molecules-26-04237-s001.zip › molecules-1250993-SI.pdf]
